# Scalable *de novo* classification of antibiotic resistance of *Mycobacterium tuberculosis*

**DOI:** 10.1093/bioinformatics/btae243

**Published:** 2024-06-28

**Authors:** Mohammadali Serajian, Simone Marini, Jarno N Alanko, Noelle R Noyes, Mattia Prosperi, Christina Boucher

**Affiliations:** Department of Computer and Information Science and Engineering, University of Florida, 1889 Museum Road, Gainesville, Florida 32611, United States; Department of Epidemiology, University of Florida, PO Box 100231, Gainesville, Florida 32601, United States; Department of Computer Science, University of Helsinki, P.O. Box 4, Helsinki 00014, Finland; Department of Veterinary Population Medicine, University of Minnesota, 1365 Gortner Avenue, St. Paul, Minnesota 55108, United States; Department of Epidemiology, University of Florida, PO Box 100231, Gainesville, Florida 32601, United States; Department of Computer and Information Science and Engineering, University of Florida, 1889 Museum Road, Gainesville, Florida 32611, United States

## Abstract

**Motivation:**

World Health Organization estimates that there were over 10 million cases of tuberculosis (TB) worldwide in 2019, resulting in over 1.4 million deaths, with a worrisome increasing trend yearly. The disease is caused by Mycobacterium tuberculosis (MTB) through airborne transmission. Treatment of TB is estimated to be 85% successful, however, this drops to 57% if MTB exhibits multiple antimicrobial resistance (AMR), for which fewer treatment options are available.

**Results:**

We develop a robust machine-learning classifier using both linear and nonlinear models (i.e. LASSO logistic regression (LR) and random forests (RF)) to predict the phenotypic resistance of *Mycobacterium tuberculosis* (MTB) for a broad range of antibiotic drugs. We use data from the CRyPTIC consortium to train our classifier, which consists of whole genome sequencing and antibiotic susceptibility testing (AST) phenotypic data for 13 different antibiotics. To train our model, we assemble the sequence data into genomic contigs, identify all unique 31-mers in the set of contigs, and build a feature matrix *M*, where *M*[*i*, *j*] is equal to the number of times the *i*th 31-mer occurs in the *j*th genome. Due to the size of this feature matrix (over 350 million unique 31-mers), we build and use a sparse matrix representation. Our method, which we refer to as MTB++, leverages compact data structures and iterative methods to allow for the screening of all the 31-mers in the development of both LASSO LR and RF. MTB++ is able to achieve high discrimination (*F*-1 >80%) for the first-line antibiotics. Moreover, MTB++ had the highest *F*-1 score in all but three classes and was the most comprehensive since it had an *F*-1 score >75% in all but four (rare) antibiotic drugs. We use our feature selection to contextualize the 31-mers that are used for the prediction of phenotypic resistance, leading to some insights about sequence similarity to genes in MEGARes. Lastly, we give an estimate of the amount of data that is needed in order to provide accurate predictions.

**Availability:**

The models and source code are publicly available on Github at https://github.com/M-Serajian/MTB-Pipeline.

## 1 Introduction

The World Health Organization (WHO) estimates that there were over 10 million cases of tuberculosis worldwide in 2019, resulting in over 1.4 million deaths, with a worrisome increasing trend yearly. The disease is usually caused by *Mycobacterium tuberculosis* (MTB), through airborne transmission. Treatment of tuberculosis is estimated to be 85% successful, however, this percentage drops to 57% with MTB that exhibits resistance to multiple antibiotic drugs (The World Health Organization 2023), for which fewer treatment options are available. Thus, the identification of antibiotic resistance is critical for patient care management, guiding antibiotic drug stewardship, and decreasing the selection of resistant strains from a public health and ecological standpoint. Typically, antibiotic resistance is measured through the Antibiotic Susceptibility Test (AST) for each antibiotic drug. DNA sequencing can be paired with AST to identify genetic differences between antibiotic-resistant and susceptible strains, both in clinical and ecological settings. Several data and model resources for MTB have been established to support research on antibiotic resistance. One of the largest is the international Comprehensive Resistance Prediction for Tuberculosis: an International Consortium (CRyPTIC), which performed whole genome sequencing for tens of thousands of MTB isolates collected from diverse locations worldwide as well as AST analysis of these isolates for over a dozen antibiotic drugs (The CRyPTIC Consortium and the 100,000 Genomes Project 2018, The CRyPTIC Consortium 2022).

Recently, the CRyPTIC database has been used for a genome-wide association study to identify oligopeptides and oligonucleotides in MTB associated with resistance to single antibiotics. These findings confirmed and expanded on prior work that identified single nucleotide polymorphisms (SNPs) that are associated with antibiotic drug resistance in MTB (Ramaswamy and Musser 1998, Riska *et al.* 2000, Rodwell *et al.* 2014). Prior methods for classifying antibiotic resistance in MTB use catalogues of genetic variants, i.e. predominantly SNPs: TBProfiler (Phelan *et al.* 2019), PhyResSE (Feuerriegel *et al.* 2015), and KvarQ (Steiner *et al.* 2014). TBProfiler combines several tools including Trimmomatic (Bolger *et al.* 2014), BWA (Li and Durbin 2009) or Bowtie 2 (Langmead and Salzberg 2012), BCFtools, and SAMtools (Danecek *et al.* 2021), to map sequence reads from whole genome sequencing to a WHO-endorsed catalogue of 17 356 MTB variants whose resistance is known. Similarly, PhyResSE is a pipeline that also uses several third-party methods, including QualiMap (García-Alcalde *et al.* 2012), SAMtools, and GATK (McKenna *et al.* 2010). Unlike TBProfiler, PhyResSE aims to identify both the lineage and resistance type from whole genome sequence data. PhyResSE aligns all the input reads to the MTB H37Rv reference genome using BWA; identifies variants using GATK; and predicts the resistance and lineage based on the found variants. KvarQ is another AMR profiler tool capable of identifying the allelic state of established polymorphisms to detect MTB AMR. This tool efficiently extracts pertinent information from each individual read, bypassing the need to map every read to a reference genome.

In addition to these, there are other methods of pairing machine learning or combinatorial approaches with variant catalogues to identify antibiotic resistance in MTB. These include Mykrobe (Bradley *et al.* 2015, Hunt *et al.* 2019), GenTB (Gröschel *et al.* 2021), and the models by Kuang *et al.* (2022). Mykrobe (Bradley *et al.* 2015, Hunt *et al.* 2019), which was initially released in 2015 and then updated in 2019, relies on the construction and analysis of a de Bruijn graph from catalogues of resistant and susceptible alleles on different genetic backgrounds, along with a set of antibiotic resistance genes. Together, they form what the authors refer to as a *reference graph*. The reference graph is compared to the de Bruijn graph of the sequence data and through this comparison, a prediction of the resistance is made based on statistical tests. The last update of Mykrobe (Hunt *et al.* 2019) included a mutation catalogue, giving greater sensitivity to detect pyrazinamide resistance; another improvement was to allow for a user-specified catalogue. GenTB is another MTB-specific AMR profiler, a machine-learning method that classifies resistance to 10 different MTB drugs using a catalogue of variant positions spanning 18 resistance-associated genetic loci. It trains both a random forest (RF) classifier and a deep neural network. A more recent work was released by Kuang *et al.*, which trains concurring machine-learning models (logistic regression (LR), RF, and convolutional neural network).

Lastly, there are general-purpose methods for classifying antibiotic resistance from either whole genome sequence data or metagenomic sequence data. Most of these methods and databases, including MEGARes (Bonin *et al.* 2023), contain curated antibiotic genes for *MTB* resistance but have not been specifically evaluated on MTB isolates. The exception to this is ResFinder (Bortolaia *et al.* 2020, Florensa *et al.* 2022), a generally purposed method that stands out as having been redeveloped and evaluated for MTB phenotypic resistance classification.

In this article, we develop a robust machine-learning classifier that predicts the phenotypic resistance of MTB for a broad range of antibiotic drugs. Our method, which we refer to as MTB++, classifies the resistance to 16 different antibiotics of an isolate based on the oligonucleotides in its whole genome sequence data. To the best of our knowledge, this is the first machine-learning method that is trained in a *de novo* fashion, meaning that it only uses the oligonucleotides and AST data for training; whereas existing classifiers—including ResFinder (Bortolaia *et al.* 2020, Florensa *et al.* 2022), GenTB (Kuang *et al.* 2022), TBProfiler (Phelan *et al.* 2019), Mykrobe (Bradley *et al.* 2015), and KvarQ (Steiner *et al.* 2014)—require prior knowledge of genetic variants that associate with MTB antibiotic resistance occurrence. It should be noted that the recent study of The CRyPTIC Consortium (2022) also considered oligonucleotides without confining interest to genetic variants, but this analysis did not include the release of a classifier. Moreover, in order to construct our classifier we consider up to three orders of magnitude more oligonucleotides in comparison to The CRyPTIC Consortium (2022) study. We enable the training of this large search space through a combination of succinct data structures and machine learning, namely LR and RF. The employment of both a large search space and a *de novo* machine-learning approach allows for novel mechanisms of resistance to be identified and for increased performance accuracy to be obtained. We evaluated our method against all available competing methods. MTB++ is able to achieve high discrimination (i.e. *F*-1 score over 80%) for 10 out of the 13 antibiotic drugs we considered that had a data balance (ratio of isolates with phenotypic resistance to total number of isolates) of over 5%. Competing methods were only able to achieve high discrimination for 5–7 of these antibiotic drugs. In addition to considering the accuracy of the classification, we identify antibiotic resistance genes containing the oligonucleotides of our models to hypothesize about novel variants associated with resistance in MTB. The trained classifier and source code to create the classifier are publicly available on GitHub at https://github.com/M-Serajian/MTB-plus-plus.

## 2 Materials and methods

### 2.1 Dataset description

We downloaded all publicly available whole genome sequencing data, and AST data for MTB from The CRyPTIC Consortium (2022). The data are available on the European Nucleotide Archive (ENA) FTP server. The whole genome sequencing data are all Illumina paired-end reads and are annotated using ERR numbers. Resistance phenotype data are annotated with ERS numbers. We matched the ERR and ERS for each isolate using a .JSON file. The ERS numbers and JSON file are available here: https://ftp.ebi.ac.uk/pub/databases/cryptic/release_june2022/. We note that isolates were removed from further consideration if either the sequence data could not be retrieved or the ERR and ERS number could not be properly matched. After the removal process, we obtained a total of 6224 isolates with complete sequence. Based on the MIC threshold used by the CRyPTIC consortium, we labeled each isolate as resistant or susceptible to 13 antibiotic drugs: amikacin (AMI), bedaquiline (BDQ), clofazimine (CFZ), delamanid (DLM), ethionamide (ETH), ethambutol (EMB), isoniazid (INH), kanamycin (KAN), levofloxacin (LEV), linezolid (LZD), moxifloxacin (MXF), rifampicin (RIF), and rifabutin (RFB). In the treatment of TB, first-line antibiotic drugs consist of EMB, INH, and RIF; second-line drugs are AMI, ETH, KAN (injectable agent), LEV, MXF, and RFB; and the last line of new and repurposed drugs are BDQ, CFZ, DLM, and LZD (The CRyPTIC Consortium 2022). The number of isolates resistant to the last line of antibiotic drugs was very low in comparison to the first two lines (e.g. <1% for BDQ). Thus, we combined some of these drugs into classes based on the classification hierarchy of Doster *et al.* (2020): (1) KAN and AMI were combined into the aminoglycoside (AMG) group, (2) RFB and RIF were combined into the rifamycin (RIA) group, and (3) MXF, LEV, and CFZ were combined into the fluoroquinolones (FQS) group. The combination process is as follows: if an isolate is phenotypically resistant to at least one of the antibiotic drugs in a group, then that isolate will be considered as resistant to that group. In Supplementary Table S2, we provide the number of isolates that were phenotypically susceptible, ambiguous, and resistant isolates, as well as, the proportion of isolates that were resistant to each antibiotic out of the total number of isolates (excluding ambiguous). The phenotypic resistance for two of the antibiotic drugs, EMB, and ETH, was “intermediate”; in those cases, we labeled the isolates as phenotypically resistant since they still show a resistant phenotype.

### 2.2 Genome assembly and feature extraction

We assembled the sequence reads for all 6224 isolates using SPAdes (version 3.15.3) (Prjibelski *et al.* 2020), and evaluated the quality of the assemblies using Quast (Gurevich *et al.* 2013). The mean and standard deviation (std) for the number of reads for each isolate is 4 397 842 and 2 685 471, respectively. The mean and standard deviation of the N50 is 96 971.55 and 44 104.37, respectively.

Next, we used all unique 31-mers from the assembled genomes as the features for our machine-learning models. Thus, we created an n×m integer matrix that stores the number of occurrences of each 31-mer in each assembled genome, where *n* is the number of unique 31-mers in all the assembled genomes, and *m* is the number of genomes. The selection of *k* being 31 was informed by prior research on prokaryotic genome assembly (Chor *et al.* 2009, Chikhi and Medvedev 2014, Prjibelski *et al.* 2020). Choosing *k*-mer sizes that are too small often results in non-informative outcomes, as such sizes are common across all isolates, while excessively large *k*-mer sizes can also be non-informative. Hence, the integer at the *i*th row and *j*th column are equal to the number of times the *i*th 31-mer occurs in the *j*th assembled genome. To build this matrix, we first constructed a perfect hash function that maps the unique 31-mers of the data to the rows of the color matrix. We used the SBWT data structure of Alanko *et al.* (2023) to implement this hash function. The hash value of 31-mer *x* is the number of distinct 31-mers in the data that are co-lexicographically smaller than *x*. For our data, the matrix has 356 359 267 rows (31-mers, *n*) and 6224 columns (genomes, *m*). Since the matrix is very large, we only store the non-zero elements to save space (ASCII format). That is, for each row in the matrix, we store a list of pairs $(g_{1},c_{1}),(g_{2},c_{2}),\ldots$, where pair $\left(g_{i},c_{i}\right)$ indicates that genome $g_{i}$ contains $c_{i}$ copies of the 31-mer of the row. These lists are built by iterating the columns (genomes) left to right and using the hash function to look up the rows of the 31-mers, creating new counter pairs $\left(g_{i},c_{i}\right)$ on demand when a *k*-mer is seen the first time in the genome, and incrementing counters in existing pairs otherwise.

### 2.3 Feature selection

The initial feature set included all 350 million 31-mers extracted *via* the SBWT, from which we removed all high- and low-frequency ones, i.e. <10 times or more than 3000 times. We then performed a multi-step procedure to select informative features, executed within the training-test splits of cross-validation and individual model selection (explained in the next section), to avoid overfitting.

First, we ranked all *k*-mers by their chi-square value, which quantifies how likely the frequency of a *k*-mer is to deviate from the null hypothesis of no difference in frequency between the resistant and susceptible class. Thus, *k*-mers exhibiting a weak association with the outcome would be at the bottom of the ranking and less likely to be selected. All the *k*-mers with a *P*-value above .05 (adjusted for multiple testing using Bonferroni criterion) were filtered out.

Second, since the chi-square test is univariate and neither recognizes correlated features (subject to confounding) nor interactions, we utilized regression methods with embedded variable shrinkage/selection and interaction identification, namely LR with the least absolute shrinkage and selection operator (LASSO) and RF, explained in the following section.

Third, we trained both LASSO LR and RF models on subsets of the whole *k*-mer space, considering an incremental number of *k*-mers according to the chi-square ranking. The best-performing model with the least number of features would be selected. In this way, a parsimonious model with fewer parameters is preferred, guaranteeing no loss in performance and helping to avoid overfitting. Operationally, we considered feature sets consisting of the $2^{i}$ top-ranked 31-mers, for all integers i ∈ [0, 19], i.e. from 1 to 524 288 features. In other words, we first considered a model with the 31-mer yielding the highest chi-square value, then considered another with the top two 31-mers, then another with the top-four, top-eight, top-16, and so forth. We continued to double the number of input features until the performance (*F*-1 score, see below) of the model did not improve over three consecutive times.

### 2.4 Model selection, cross-validation, and performance measures

We trained both LASSO LR and RF models for each feature subset and for each antibiotic drug, i.e. a total of 16 LR models and 16 RF models. LR and RF were chosen because they complement each other in terms of complexity, performance, and interpretability trade-offs (Bishop and Nasrabadi 2006). LR is a parametric algorithm that models the probability of an outcome (i.e. being resistant to an antimicrobial) as a linear function of input features incorporated into a logistic function. In the absence of causal assumptions and/or correlation structures, the coefficients of LR are relatively simple to interpret as weights contributing to increased or decreased likelihood of the outcome. It is worth noting that the coefficients from LASSO are rescaled and cannot be directly transformed into odds ratios and should not be used as a measure of absolute magnitude for variable importance, but we used them only relative to each other in the same model fit with the same shrinkage value. More importantly, LR struggles with nonlinear relationships, unless they are specifically encoded as additional input variables, e.g. tensor splines. In contrast, RF is fully nonparametric and nonlinear. RF combines multiple decision trees, each trained on a bootstrapped subset of the data and on randomly selected feature subsets, to make class predictions through voting. Feature importance in RF can be determined in multiple ways; one is to look at the average reduction in impurity across trees. Due to its nonlinear property, RF is more flexible and capable of capturing interactions between features that the LR cannot directly handle. RF exhibits higher complexity due to multiple decision trees, which could be prone to overfitting, but the problem is mitigated through bootstrapping and random feature selection. RF generalizes well and often delivers strong performance across tabular datasets in computational biology, even when compared to deep learning (Grinsztajn *et al.* 2022). For this work, LASSO LR and RF are not combined in an ensemble, but assessed independently, selecting one model for each antibiotic.

To assess the performance of our models and compare it to the previous models, we use *F*-1 score, which is the harmonic mean of precision and recall, i.e. $2\cdot \frac{\text{precision}\cdot \text{recall}}{\text{precision}\;+\;\text{recall}}$. The *F*-1 score is robust with respect to data imbalance compared to other evaluation metrics. Here, a sample is considered positive for a class if it is resistant to that class. We also report the precision and recall separately.

To assess extra samples, i.e. generalization and performance, we use 5-fold cross-validation. As mentioned above, the multi-step feature optimization procedure was run within each training fold to avoid overfitting. In terms of hyperparameter optimization, for the LASSO regression, we used the default penalty parameter because a full-blown optimization would not be computationally feasible, requiring a complete refit of the model with the whole incremental feature selection procedure for each different penalty value. Thus, the LASSO penalty might be suboptimal, yet we consider this as an acceptable choice, as it would only underestimate the performance in cross-validation. We anticipate that the results will show that even with underestimation our model is superior to the competitors. For the RF, instead, we optimized the number of trees—since the individual trees are fit incrementally—up to 250, stopping at 150 in the absence of a significant performance increase.

## 3. Results

### 3.1 Performance of MTB++ *via* cross-validation

**Figure 1.**
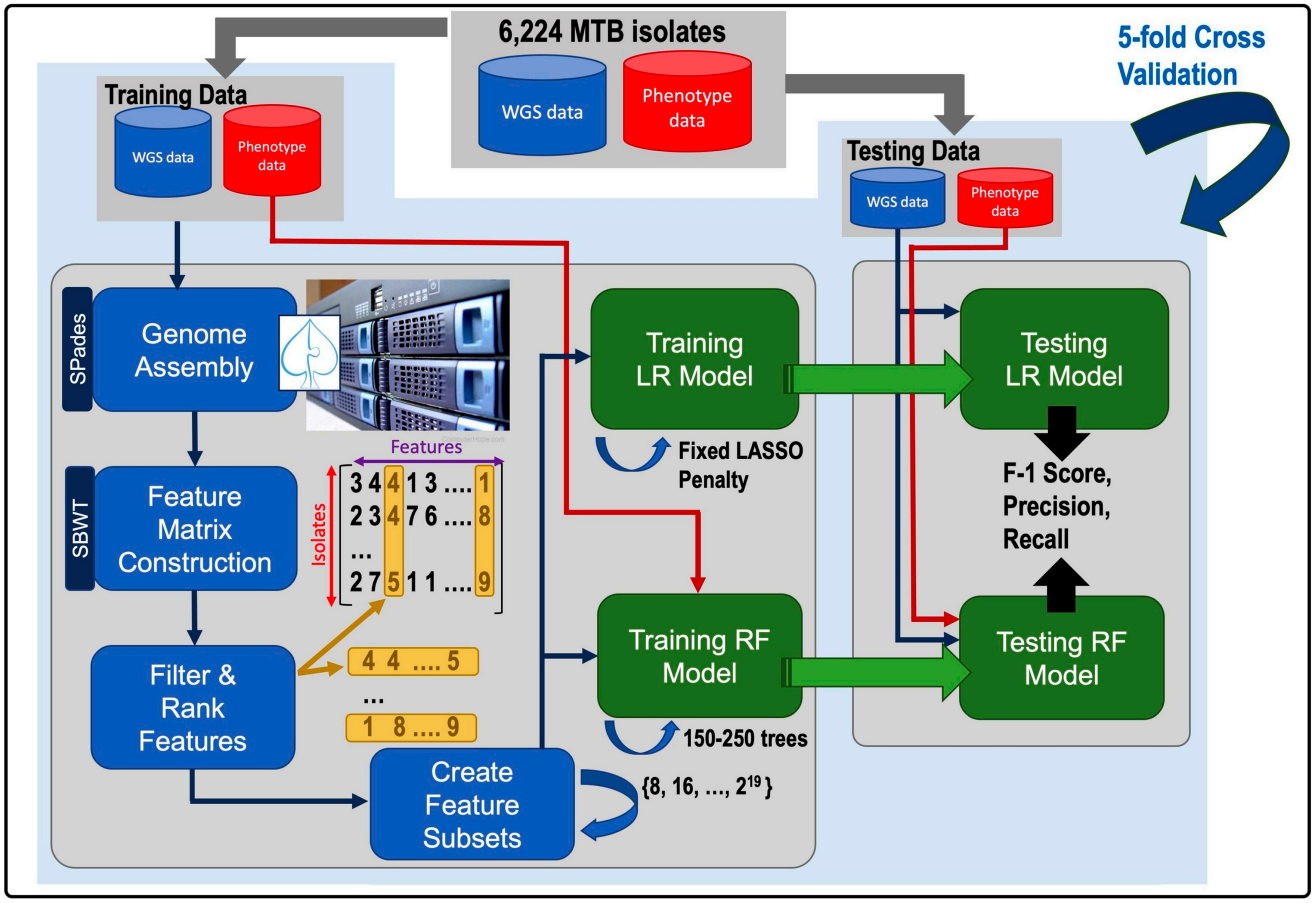
Overview of MTB++. We downloaded all publicly available data available through The CRyPTIC Consortium (2022). Split the data into training and testing datasets. We trained both a linear regression (LR) and random forest (RF) model on each dataset. The training data were assembled using SPAdes (Bankevich *et al.*, 2012), and then all unique 31-mer were extracted in order to create a feature matrix for training the models. Five-fold cross-validation was used to evaluate the trained models and calculate the *F*-1 score.

We give an overview of our methods in Fig. 1. We downloaded all publicly available whole genome sequencing data, and phenotypic data for MTB from The CRyPTIC Consortium (2022). We give the number of isolates that were deemed to be susceptible, resistant, and ambiguous to each antibiotic drug based on the CRyPTIC AST in Supplementary Table S2. The percentage that is resistant is given since it has a significant effect on the performance of the methods we evaluated. We then assembled the sequence data from all isolates in CRyPTIC that have both phenotypic and genomic data and identified the set of all unique 31-mers from these contigs extracted as features for classification. This resulted in 6224 sets of contigs, 350 million 31-mers before filtering and close to 17 million 31-mers after filtering. Five-fold cross-validation was used to evaluate our methods. We divided the data into five equal parts. In each of the five steps, one part was used to test, and the other four parts were for training our models and ranking features. This was done for both LR and RF models. We report the *F*-1 scores in Table 1. In addition, we report the *F*-1 scores for MTB++ LR and MTB++ RF separately in Supplementary Table S3. In addition to the *F*-1 score, we illustrate the receiver operating characteristic (ROC) curve for each antibiotic drug in Supplementary Fig. S4. Our method achieved an *F*-1 score >90% in four classes, and another eight classes had an *F*-1 score >75%.

**Table 1.** Comparison of *F*-1 scores for MTB++ and other methods.^a^

R. Class	Balance (%)	MTB++	ResFinder	TBProfiler	Mykrobe	KVarQ
INH	48.96	**95.83** (0.01)	94.57	95.04	95.15	93.04
RIA	42.69	**92.75** (0.01)	N/A	N/A	N/A	N/A
RIF	41.60	**94.57** (0.11)	94.37	94.28	94.17	93.51
RFB	38.83	**90.65** (0.02)	N/A	N/A	N/A	N/A
EMB	34.49	**86.21** (0.02)	86.35	**87.3**	86.63	78.95
FQS	22.67	**82.02** (0.10)	81.84	81.94	N/A	78.19
ETH	21.21	**76.43** (0.01)	72.75	76.11	75.27	N/A
LEV	19.6	**87.43** (0.08)	N/A	86.29	87.36	N/A
MXF	15.81	**80.77** (0.01)	N/A	80.18	80.15	N/A
AMG	10.2	**75.38** (0.03)	N/A	72.95	N/A	N/A
KAN	9.21	**80.37** (0.01)	77.38	78.01	78.63	N/A
AMI	7.83	**82.12** (0.00)	80.67	80.47	80.04	N/A
CFZ	3.98	**13.82** (0.01)	0.73	2.86	N/A	N/A
DLM	1.79	**12.78** (0.01)	N/A	1.77	0	N/A
LZD	1.27	**21.74** (0.01)	31.19	**32.73**	27.18	N/A
BDQ	0.72	**8.51** (0.00)	2.82	5.19	N/A	N/A

a Our performance is demonstrated by the mean and (std) of the *F*1-score in cross-validation. The largest F1-score is in bold. For some of the competing methods, some of our drugs in this study were not covered, and those cases are demonstrated using N/A. The balance of the data is reported as a percentage and is equal to the number of resistant isolates divided by the number of resistant isolates plus the number of susceptible isolates. The values for the number of resistant and susceptible isolates are given in Supplementary Table S2.

We compared the performance of MTB++, ResFinder (Bortolaia *et al.* 2020), Mykrobe (Hunt *et al.* 2019), KvarQ (Steiner *et al.* 2014), and TBProfiler (Coll *et al.* 2015, Phelan *et al.* 2019). We note that we were unable to compare against PhyResSE (Feuerriegel *et al.* 2015), GenTB (Gröschel *et al.* 2021), and the method of Kuang *et al.* (2022) (Kuang *et al.* (2022) do not provide a trained classifier. All methods were run with their default settings. PhyReSE is only provided as a web interface that takes in a single genome at once and is unable to handle large batch files. GenTB failed with source code errors. The issue was posted to the GenTB GitHub: https://github.com/farhat-lab/gentb-snakemake/issues/6.). MTB++ had the highest *F*-1 score for 13 of the 16 antibiotic drugs evaluated and achieved a performance of >90% for two antibiotics (RIA and RFB) in which the competing methods were unable to make any prediction. KVarQ was only able to perform a prediction for four out of the 16 antibiotic drugs. Mykrobe could provide predictions for nine of the 16 antibiotics but had lower performance than MTB++ in eight of those nine antibiotics. Mykrobe outperformed MTB++ for LZD, yet the *F*-1 score of both methods was <28%. TBProfiler and ResFinder were the most competitive but ResFinder only had acceptable performance (i.e. >70 %) in seven of 16 antibiotics. TBProfiler achieved acceptable performance on 10 out of the 16 antibiotics but had a lower *F*-1 score than MTB++ on nine out of those 10 antibiotics. In summary, MTB++ had superior performance than all competing methods for almost all antibiotic drugs; the only ones for which the competing methods outperformed our method were EMB and rare antibiotic drugs which have highly unbalanced data (the ratio of isolates with resistant phenotype to total number of isolates is below 40%). Moreover, all methods were unable to achieve acceptable performance on four of the 16 antibiotics, where the data were highly unbalanced; these antibiotics are BDQ, LZD, DLM, and CFZ. Each of these antibiotic drugs is reserved for the use of multi-drug-resistant MTB in order to ensure judicious use and because of their numerous side effects, including drug interactions and even death as in the case of BDQ (Cox and Laessig 2014).

Supplementary Table S4 compares the performance of MTB++ against all competing methods (Resfinder, TBProfiler, Mykrobe, and KVarQ) across various resistance classes using precision and recall metrics. MTB++ shows superior performance with a balance of high precision and recall, suggesting its effectiveness in accurately identifying and comprehensively detecting INH resistance. Mykrobe closely follows MTB++ in precision but with a slightly lower recall, indicating a minor shortfall in detecting all positive cases. KVarQ, while having the highest precision, has a significantly lower recall, which could imply missed detection. Resfinder and TBProfiler show slightly lower precision than MTB++ but maintain competitive recall rates. With respect to RIF resistance, MTB++ demonstrates strong performance, closely matched by TBProfiler in recall but leading in precision. KVarQ and Mykrobe present lower precision and recall compared to MTB++, indicating a reduced ability to accurately detect RIF resistance. Resfinder has a lower precision but a higher recall, suggesting a tendency to identify more positive cases at the expense of accuracy. For EMB, FQS, and ETH, MTB++ generally shows strong precision, indicating its accuracy, but its recall varies, suggesting differences in its ability to detect all cases across different resistance classes. Notably, for CFZ and DLM, MTB++ has significantly higher precision but very low recall, highlighting a potential limitation in these specific contexts. Other methods have varying performances across different drugs, with several N/A entries indicating a lack of data or applicability in those cases. Overall, MTB++ stands out for its balance of precision and recall across most resistance classes, suggesting it as a reliable method for detecting various types of drug resistance. Its consistently high precision across all classes indicates a strong accuracy in identifying true resistance cases. Other methods vary in their precision and recall, with some (like TBProfiler and Mykrobe) closely competing with MTB++ in certain classes but not as consistently across the board. The lack of data for certain methods against specific drugs (indicated by N/A) highlights gaps in their applicability or testing.

This comparison underscores the importance of choosing the right diagnostic method based on the specific resistance class being tested and the balance of precision and recall that is desired.

### 3.2 Feature analysis

**Figure 2.**
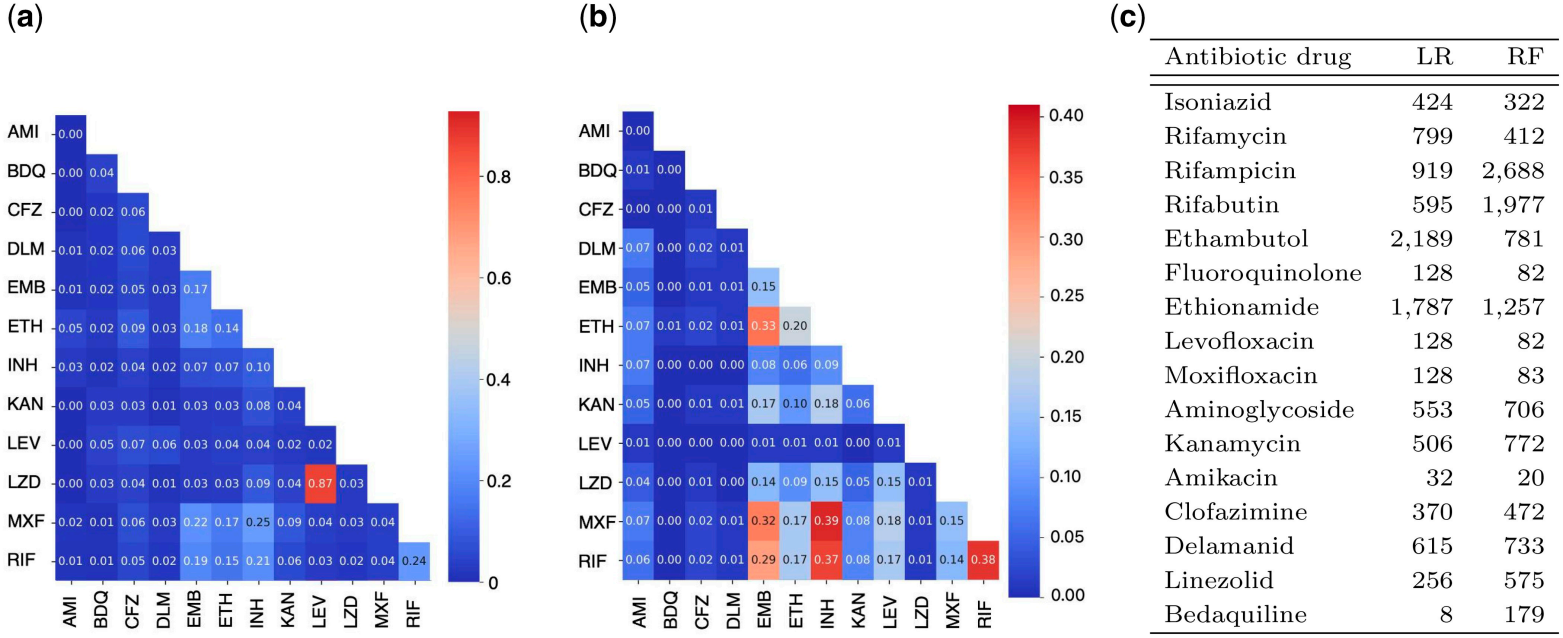
On the left (a) is a heatmap illustrating the Jaccard index for shared 31-mers between all pairs of antibiotic drugs, normalized by the total number of 31-mers in the union of features for the two antibiotic drugs. In the center (b) is a heatmap that gives the ratio of isolates resistant to two distinct antibiotic drugs to the total number of isolates. On the right (c) is a table giving the number of features (31-mers) for each antibiotic drug needed to optimize the *F*1-score of the LR and RF models.

One of the advantages of MTB++ is that it is *de novo*, meaning that it uses no prior knowledge about the genetic variations that have been confirmed to be associated with MTB antibiotic resistance. Our method begins by considering all unique 31-mers, resulting in over 350 million 31-mers and filters for those that occur sparingly or ubiquitously. From the set of frequency-filtered 31-mers, we trained the models according to the multi-step procedure, i.e. chi-square ranking and incremental feature selection to maximize *F*-1 score. The number of features that had a non-zero coefficient from these models is reported in Table demonstrated in Fig. 2c.

There was high redundancy between the 31-mers for each antibiotic, as highlighted by the number of features with non-zero coefficients relative to the total feature count selected by the LASSO LR. It is likely that adjacent 31-mers in resistant genes convey similar or redundant information (belonging to the same mechanism of resistance), thus LASSO LR is likely to select one representative feature, reducing model complexity and enhancing model interpretability. The 31-mer importance in RF (Gini impurity measure) also shows a skewed distribution, with fewer highly important 31-mers compared to the whole feature space. Correlated features in RF that have high predictive value tend to have lower importance because they have the same likelihood of being selected in a tree than their correlates, thereby decreasing their impact overall. However, there is not a single representative selection like in the LASSO LR. Thus, RF may include more feature variety, yet it is more difficult to assess individual importance. This fact underscores the importance of considering both methods when comparing predictive features among different antibiotics. Accordingly, we combined the top 31-mers selected by either RF or LASSO LR.

### 3.3 Alignment of features to AMR database

In order to further evaluate and use the model, we extracted all features that had a non-zero coefficient from the model that obtained the optimal *F*-1 score for each antibiotic drug and aligned these features to MEGARes 3.0 (Bonin *et al.* 2023) database using BWA (Li and Durbin 2009) with the restriction that we only considered exact matches. MEGARes has over 8000 AMR genes with less than two dozen of these being MTB-specific antibiotic resistance genes. MEGARes also contain metal resistance and virulence factors. After alignment, we omitted any AMR genes where there were <10 31-mers aligned to it; this was done to remove any spurious results. Supplementary Tables S5 and S6 give the MEGARes genes by antibiotic drugs for each MEGARes resistance class. Supplementary Table S5 is restricted to antibiotic drugs for which the balance of the data is at least 15%. Supplementary Table S6 is restricted to antibiotic drugs for which the balance of the data is at most 11%. In order to further contextualize our results, we first considered the possible multi-drug resistance of the isolates. Figure 2b illustrates the frequency of resistance to two different antibiotic drugs. Complementary to this, we also calculated the Jaccard similarity index of the set of features for each pair of antibiotic drugs. The Jaccard similarity compares members for two sets to determine which members are shared and which are distinct. Figure 2b gives an illustration of Jaccard similarity.

Figure 2b illustrates that there is a high frequency of co-resistance between RIA and RIF (42%), RIA and RFB (39%), and RIF and RFB (38%). It is worth noting that these antibiotic drugs are associated with a larger family of resistance: RIAs, which is a first-line therapy for mycobacterial infections. INH resistance was also associated with resistance to RIA, RIF, and RFB. However, there was not as strong of a correlation with the Jaccard index between these classes of resistance as can be seen in Fig. 2a. Our results in Supplementary Table S5 are reflective of this similarity. INH, RIA, RIF, and RFB had exact alignments to several of the same genes in MEGARes, including MEG_8171 (katG), MEG_6144 (rpsL), MEG_3237 (GyrA), MEG_2710, MEG_2711 (embB), MEG_7259 (remB), MEG_6090, MEG_6134 (rpoB). RFB is uniquely associated with MEG_3180 (GyrA), which is associated with fluoroquinolone resistance (Weigel *et al.* 1998).

Other pairs of antibiotic drugs that had high Jaccard similarity were: LEV–fluoroquinolone, MXF–fluoroquinolone, and MXF–LEV. LEV and MXF are frequently classified as FQS, which validates our model. This relationship is further reflected in Supplementary Table S5 as MEG_2337, which is a fluoroquinolone antibiotic resistance gene, has the largest number of exact alignments to LEV, fluoroquinolone, and MXF.

There were several AMR genes that were unique to specific antibiotic drugs. These associations validate our model and demonstrate the possible novel mechanisms of resistance. MEG_2712 was uniquely associated with EMB. This AMR gene is defined as being in the EMB class in MEGARes and targets arabinosyltransferase. Hence, this result validates our findings. More surprisingly, EMB was uniquely associated with MEG_2653, which corresponds to copper resistance in MEGARes. MEG_8079, which is classified as an MTB-specific AMR gene in MEGARes, also targets arabinosyltransferase. This gene was uniquely associated with RIF. Interestingly EMB and RIF are frequently combined for the treatment of MTB so it is conceivable that there is a region in MEG_8079 that is encoding for RIF resistance or this was due to multi-drug resistance caused by the concurrent treatment with EMB and RIF. Lastly, EMB was also associated with MEG_1490, which is defined as a cationic antibiotic peptide (CAMP) resistance gene in MEGARes.

In addition, we again extracted all features that had non-zero coefficients from the model that obtained the optimal *F*-1 score for each antibiotic drug and considered their alignment to the MTB reference genome (H37Rv, NCBI Reference Sequence: NC_000962.3). Again, we used BWA (Li and Durbin 2009) for alignment, configuring the parameters to guarantee that only exact alignments are identified and retrieved. Next, BEDTools (version 2.30.0) (Quinlan and Hall 2010) is used to intersect BAM files with the MTB GFF file (NCBI RefSeq assembly: GCF_000195955.2) for genomic feature analysis. Moreover, we omitted any AMR genes where there were less than ten 31-mers aligned to it; this was done to remove any spurious results. We compared our results with the results from the CRyPTIC consortium to identify novel genomic regions. The GTF file was used to identify the genes. Supplementary Table S7 gives the results of this experiment. The results show high concordance with the results from the CRyPTIC consortium but this was consistent across the antibiotic drugs considered. For example, the majority of the associations seen for INH, RIF, LEV, MXF, KAN, and AMI were previously reported by the CRyPTIC consortium. In particular, gyrA is an MTB gene that was frequently associated with multiple antibiotics by the CRyPTIC consortium and this study. The gyrA gene is also significant in the context of antibiotic resistance because mutations in gyrA can lead to resistance against FQS, a class of antibiotics that target DNA gyrase and interfere with the enzymes’ ability to introduce supercoils into DNA, thereby inhibiting bacterial DNA replication and transcription. Mutations in the gyrA gene alter the enzyme’s structure in a way that reduces the drug’s binding affinity, allowing the bacterium to survive in the presence of the antibiotic. This mechanism is a common cause of fluoroquinolone resistance in MTB and other bacterial pathogens. Other genes that were found to have associations in both studies include rpoB and rpoC, which encode subunits of RNA polymerase, which is an enzyme complex responsible for transcribing DNA into RNA. RNA polymerase plays a central role in the process of gene expression, enabling the synthesis of messenger RNA (mRNA), which is subsequently translated into proteins. The rpo genes are essential for the viability of the bacterium and its ability to cause disease. In addition to validating previous findings, we also demonstrate some novel associations.

For example, the PE_PGRS family of genes was found to have associations with LZD, DLM, Ethnomadie, RFB, and RIF. This family of genes is characterized by the presence of a Pro-Glu (PE) motif at their N-terminus and a polymorphic GC-rich repetitive sequence (PGRS) domain. This family is highly represented within the MTB genome and is thought to play significant roles in the bacterium’s pathogenesis, immune evasion, and interaction with the host immune system. The precise roles of many PE_PGRS proteins are yet to be fully determined. The diversity of these proteins presents challenges for developing vaccines, necessitating immune responses that can effectively target a wide array of variants. Notably, DLM is linked to four of these genes, while BDQ is linked to two. Both drugs are mainly utilized in treating multi-drug-resistant TB (MDR-TB) and extensively drug-resistant TB (XDR-TB). Therefore, understanding the connection between these genes and the mentioned antibiotics could be key in unraveling MTB pathogenesis and advancing vaccine development.

### 3.4 Analysis of last resort antibiotic drugs

**Figure 3.**
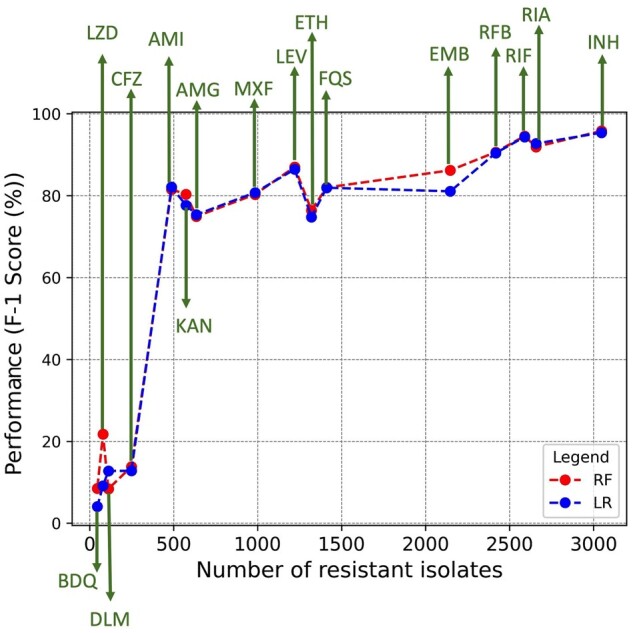
Illustration of the relationship between the optimal *F*-1 score of random forests and logistic regression (*y*-axis) and the number of resistant isolates, for each antibiotic (*x*-axis).

As shown in Table 1, the performance of MTB++ was highly dependent on the balance of the data. MTB++ had an *F*-1 score of over 90% for antibiotic drugs that had balanced data, i.e. >38%. When the balance was below 5% all the models struggled with adequate performance; for CFZ, DLM, LZD, and BDQ all the models had an *F*-1 score of 33% or below. Next, we went beyond the balance of the data and considered the number of isolates that were deemed to be phenotypically resistant in the CRyPTIC dataset. Hence, Fig. 3 illustrates the number of isolates versus the *F*-1 score for each antibiotic drug. When the number of resistant isolates was at least 500 there was a sharp increase in the performance of both the RF and LR models. In the case of antibiotic drugs representing resistance to antibiotics prescribed as a last resort (i.e. BDQ and LZD) that had a balance of <1.3% (or <80 resistant isolates), the performance was degraded as the model struggled to find enough significant features to distinguish the resistant isolates. Notwithstanding, it should be noted that even for rare antibiotic drugs—where the balance is <5%—our model achieved an *F*-1 score between 8% and 22%. This implies that our model is finding oligonucleotides that are associated with the various antibiotic drugs and could be related to new and/or undiscovered mutations that are not among currently known variants. Note that the estimation provided across all antibiotics is more comprehensive than one made on a single drug because it also considers the variance due to different class imbalance and genetic variability (Christodoulou *et al.* 2021).

From Supplementary Table S6, we show that AMI, KAN, and AMG have a considerable number of common antibiotic resistance genes and that most of these antibiotic resistance genes belong to the AMG class in MEGARes. MEG_5 was uniquely found for these three antibiotic drugs. AMI and KAN belong to the same family of antibiotics known as AMGs so this association helps validate our model.

DLM and LZD had a very low number of phenotypically resistant isolates, and thus, had a data balance of 1.79% and 1.27%, respectively; only BDQ had a lower balance (i.e. 0.72%). DLM had an association with a multi-drug antibiotic resistance gene (MTRAD) and a biocide resistance antibiotic resistance gene (MDTK). Further investigation into these associations is warranted but appears to be reasonable given that these antibiotics are considered last-resort drugs used for multi-drug-resistant MTB. Figure 2b demonstrates that very few isolates displayed co-resistance with DLM. Moreover, there were no other antibiotic drugs that had a Jaccord index of more than 0.03 with DLM.

LZD resistance was associated with MEG_1490, an antibiotic resistance gene linked to CAMP resistance that was also associated with resistance to ETH. It should be noted that there were very few isolates that had phenotypic resistance to both ETH and LZD; as shown in Fig. 2b. Phenotypic resistance to LZD was uniquely associated with the oxazolidinone antibiotic resistance class in MEGARes (MEG_8670). Oxazolidinones, a new chemical class of synthetic antibiotics, has a unique mechanism of action that involves the inhibition of bacterial protein synthesis. LZD drugs belong to the oxazolidinone class of antibiotics (Hashemian *et al.* 2018) so this finding appears to also validate the model.

### 3.5 Computational resources required by MTB++

We note that the trained MTB++ classifier takes as input a genome assembly and produces a prediction of the resistance profile. Using one of the testing datasets (consisting of 1200 isolates), we calculated (a) the number of contigs and (b) the total size of the assembly, and compared these values to the peak memory usage and CPU time using AMD EPYC 75F3 processor. All datasets ran in <50 CPU seconds and <200 MB of memory. Thus, MTB++ should be able to run on any modern desktop in less than a minute.

Training the classifier required a significant amount of computing resources and CPU time. Thus, we provide the trained classifier as a publicly available resource. For completeness, we briefly describe the computing resources for feature selection and classifier training. The feature selection requires three steps: constructing the SBWT from the assemblies of the whole genome sequence data of the isolates, transforming the SBWT to a format for machine learning (.npy format), and ranking the features. The resources required for SBWT are documented in Alanko *et al.* (2023) study. The second step required at most 24 h of wall-time on a cluster with 12 nodes, each having at most 200 GB of memory. The last step required was required for each antibiotic and fold for the cross-validation. For each individual antibiotic drug and fold, this last step required at most 12 h of wall-time on a cluster with 12 nodes each having 200 GB. Training the classifiers required at most 12 h of wall-time on a cluster with 16 nodes each having 120 GB of memory.

## 4 Discussion

The performance of MTB++ suggests that accurate performance of antibiotic resistance of MTB can be achieved for a wider range of antibiotic drugs. This is critically important since competing methods make accurate predictions for a more limited set of antibiotic drugs. The key strength of our methodology lies in its capacity to predict without relying on pre-existing knowledge of genetic variants linked to resistance. By not depending on prior information about known resistant variants, our method holds the potential to uncover and understand new genetic changes that could play a significant role in resistance, broadening our comprehension in this field. Our *de novo* construction of the feature set requires us to consider three orders of magnitude more features than prior studies before filtering and over two times more features after filtering. We note that after filtering, the feature matrix is over 1.2 terabytes in size. The space- and memory-efficient method of Alanko *et al.* (2023) study for building and storing the feature matrix allows us to build, train, and analyze a classifier on a feature matrix of this magnitude. This area of combining succinct data structures with machine-learning classification is still in a nascent stage but as biological datasets increase in size, we believe this will quickly become an area that requires further exploration. For instance, parallelization and scaling of cross-validation procedures that incorporate multi-step feature and model selection procedures with such large datasets is a priority. One limitation of our approach was that we could not perform a comprehensive hyperparameter optimization for the LASSO LR, and could not test ensemble classifiers due to computational burden, despite the preprocessing advantage obtained by the SBWT.

Finally, we note that classifiers like MTB++, which limit their analysis to genetic markers for detecting antibiotic resistance, overlook the complex genomic mechanisms that govern AMR. This includes conditional gene expression, the influence of genomic context, and the synergistic effects of multiple genes. Additionally, they do not account for horizontal gene transfer, post-translational modifications, and epigenetic factors, nor do they consider phenotypic heterogeneity where bacteria express resistance under specific conditions undetectable by genetic analysis alone. These gaps highlight the need for a more nuanced understanding of the relationship between AMR genotypes and phenotypes. Recognizing these limitations is crucial for refining models and developing more comprehensive strategies to accurately capture the complexity of resistance in MTB.

## Supplementary Material

btae243_Supplementary_Data

## Data Availability

The models and source code are publicly available at https://github.com/M-Serajian/MTB-plus-plus.
