# Supplementary material for: Scalable de novo classification of antibiotic resistance of Mycobacterium tuberculosis
Source: Bioinformatics. 2024 Jun 28;40(Suppl 1):i39–47. doi: 10.1093/bioinformatics/btae243 (PMC11211809; doi:10.1093/bioinformatics/btae243)
Supplement: btae243_Supplementary_Data [file btae243_supplementary_data.pdf]

Supplement

	Resistance Class	No. Resistant (%)	No. Susceptible	No. Ambiguous
First-line	Isoniazid	3,047 (49.00%)	3,119	58
	Rifampicin	2,589 (41.96%)	3,581	54
	Ethambutol	2,147 (34.50%)	4,036	41
Second-line	Rifabutin	2,417 (39.15%)	3,757	50
	Ethionamide	1,320 (21.20%)	4,816	88
	Levofloxacin	1,220 (19.60%)	4,950	54
	Moxifloxacin	984 (15.81%)	5,196	44
	Kanamycin	573 (9.21%)	5,565	86
	Amikacin	487 (7.82%)	5,682	55
Last-resort	Clofazimine	248 (3.98%)	5,882	94
	Delamanid	111 (1.78%)	5,989	124
	Linezolid	79 (1.27%)	6,116	29
	Bedaquiline	45 (0.72%)	6,059	120

Table 2. The number of genomes that are deemed to be susceptible or resistant for each drug or resistance type. The total number of genomes is 6,224. The percentage of resistant isolates is calculated as the number of resistant isolates divided by the number of resistant isolates plus the number of susceptible isolates.

Resistance Class	Balance	MTB++ RF	MTB++LR	ResFinder	TBProfiler	Mykrobe	KVarQ
INH	48.96	**95.83** (0.01)	95.39 (0.01)	94.57	95.04	95.15	93.04
RIA	42.69	91.93 (0.01)	**92.75** (0.11)	N/A	N/A	N/A	N/A
RIF	41.60	**94.57** (0.11)	94.37 (0.00)	94.37	94.28	94.17	93.51
RFB	38.83	**90.65** (0.02)	90.42 (0.00)	N/A	N/A	N/A	N/A
EMB	34.49	86.21 (0.02)	81.08 (0.02)	86.35	**87.3**	86.63	78.95
FQS	22.67	**82.02** (0.10)	81.96 (0.08)	81.84	81.94	N/A	78.19
ETH	21.21	**76.43** (0.01)	74.80 (0.01)	72.75	76.11	75.27	N/A
LEV	19.6	**87.43** (0.08)	86.45 (0.01)	N/A	86.29	87.36	N/A
MXF	15.81	80.32 (0.00)	**80.77** (0.01)	N/A	80.18	80.15	N/A
AMG	10.2	74.95 (0.12)	**75.38** (0.03)	N/A	72.95	N/A	N/A
KAN	9.21	**80.37** (0.01)	77.68 (0.01)	77.38	78.01	78.63	N/A
AMI	7.83	81.54 (0.00)	**82.12** (0.00)	80.67	80.47	80.04	N/A
CFZ	3.98	**13.82** (0.01)	12.81 (0.01)	0.73	2.86	N/A	N/A
DLM	1.79	8.40 (0.01)	**12.78** (0.01)	N/A	1.77	0	N/A
LZD	1.27	21.74 (0.01)	9.17 (0.01)	31.19	**32.73**	27.18	N/A
BDQ	0.72	**8.51** (0.00)	4.08 (0.00)	2.82	5.19	N/A	N/A

Table 3. Comparison between MTB++'s random forest (RF) and linear regression (LR) with other methods. Our performance is demonstrated by the mean and (std) of the F1 score in cross-validation. For some of the competing methods, some of our drugs in this study were not covered, and those cases are demonstrated using N/A.

Resistance Class	MTB++		Resfinder		TBProfiler		Mykrobe		KVarQ	
	Prec	Recall	Prec	Recall	Prec	Recall	Prec	Recall	Prec	Recall
INH	97.86	93.93	96.08	93.11	96.39	93.73	96.92	93.44	97.69	88.81
RIA	94.20	91.09	N/A	N/A	N/A	N/A	N/A	N/A	N/A	N/A
RIF	95.22	93.68	92.74	96.06	91.92	96.76	91.79	96.68	92.65	94.40
RFB	90.14	91.11	N/A	N/A	N/A	N/A	N/A	N/A	N/A	N/A
EMB	86.10	86.30	85.80	86.91	86.45	88.17	87.73	85.56	89.59	70.56
FQS	90.64	74.84	86.57	77.60	86.69	77.68	N/A	N/A	87.95	70.38
ETH	77.71	75.98	77.87	68.26	75.70	76.52	77.82	72.88	N/A	N/A
LEV	89.51	85.32	N/A	N/A	84.69	87.95	86.07	88.69	N/A	N/A
MXF	76.27	86.03	N/A	N/A	71.25	91.67	72.87	93.09	N/A	N/A
AMG	84.87	67.50	N/A	N/A	92.29	60.31	N/A	N/A	N/A	N/A
KAN	91.24	72.01	72.96	82.37	73.12	83.60	75.65	81.85	N/A	N/A
AMI	86.24	78.38	88.48	74.13	83.31	77.82	84.31	76.18	N/A	N/A
CFZ	68.05	7.66	4.17	0.40	12.79	1.61	N/A	N/A	N/A	N/A
DLM	98.01	6.90	N/A	N/A	53.10	0.90	N/A	N/A	N/A	N/A
LZD	72.77	12.60	56.64	21.52	58.11	22.78	58.31	17.72	N/A	N/A
BDQ	100.00	4.44	3.86	2.22	6.24	4.44	N/A	N/A	N/A	N/A

Table 4. Comparison of the precision and recall between MTB++ with other methods. Our performance is demonstrated by the precision and recall in cross-validation. For some of the competing methods, some of our drugs in this study were not covered, and those cases are demonstrated using N/A.

Antibiotic drug	MEGARes Resistance Class	MEGARes Accession
Isoniazid	MTB-specific	MEG_2710, MEG_2711, MEG_3446, MEG_8171
	Rifampin	MEG_6090, MEG_6134, MEG_7259
	Aminoglycoside	MEG_6144
	FQS	MEG_3237
RIA	MTB-specific	MEG_2710, MEG_2711, MEG_3446, MEG_8171
	Rifampin	MEG_6090, MEG_6134, MEG_7259
	AMG	MEG_6144, MEG_8680
	FQS	MEG_3237
Rifampicin	MTB-specific	MEG_2710, MEG_2711, MEG_3446, **MEG_8079**, MEG_8171,
	Rifampin	MEG_6090, MEG_6134, MEG_7259
	AMG	MEG_3, MEG_11, MEG_6144, MEG_8680
	FQS	MEG_3237
Rifabutin	MTB-specific	MEG_2710, MEG_2711, MEG_3446, MEG_8171
	Rifampin	MEG_6090, MEG_6134, MEG_7259, MEG_8675
	AMG	MEG_3, MEG_6144
	FQS	**MEG_3180**, MEG_3237
Ethambutol	MTB-specific	MEG_2710, MEG_2711, **MEG_2712**, MEG_3446, MEG_8171,
	Rifampin	MEG_6090, MEG_6134, MEG_7259, MEG_8675
	Aminoglycoside	MEG_11, MEG_12, MEG_6144
	FQS	MEG_3237
	Copper Resistance	**MEG_2653**, **MEG_2654**
FQS	FQS	MEG_3237
Levofloxacin	FQS	MEG_3237
Moxifloxacin	FQS	MEG_3237
Ethionamide	MTB-specific	MEG_2710, MEG_2711, MEG_3446, **MEG_7973**, MEG_8171
	Rifampin	MEG_6090, MEG_6134, MEG_7259, MEG_8675
	FQS	MEG_3237
	AMG	MEG_3, MEG_9, MEG_11, MEG_6144, **MEG_6145**, MEG_8078, MEG_8680
	Cationic AMR peptides	MEG_1490

Table 5. Classes of resistance found in the MEGARes database using BWA for all antibiotic drugs where the balance of the data is at least 15%. The bold genes in the table are uniquely found for that specific class of resistance.

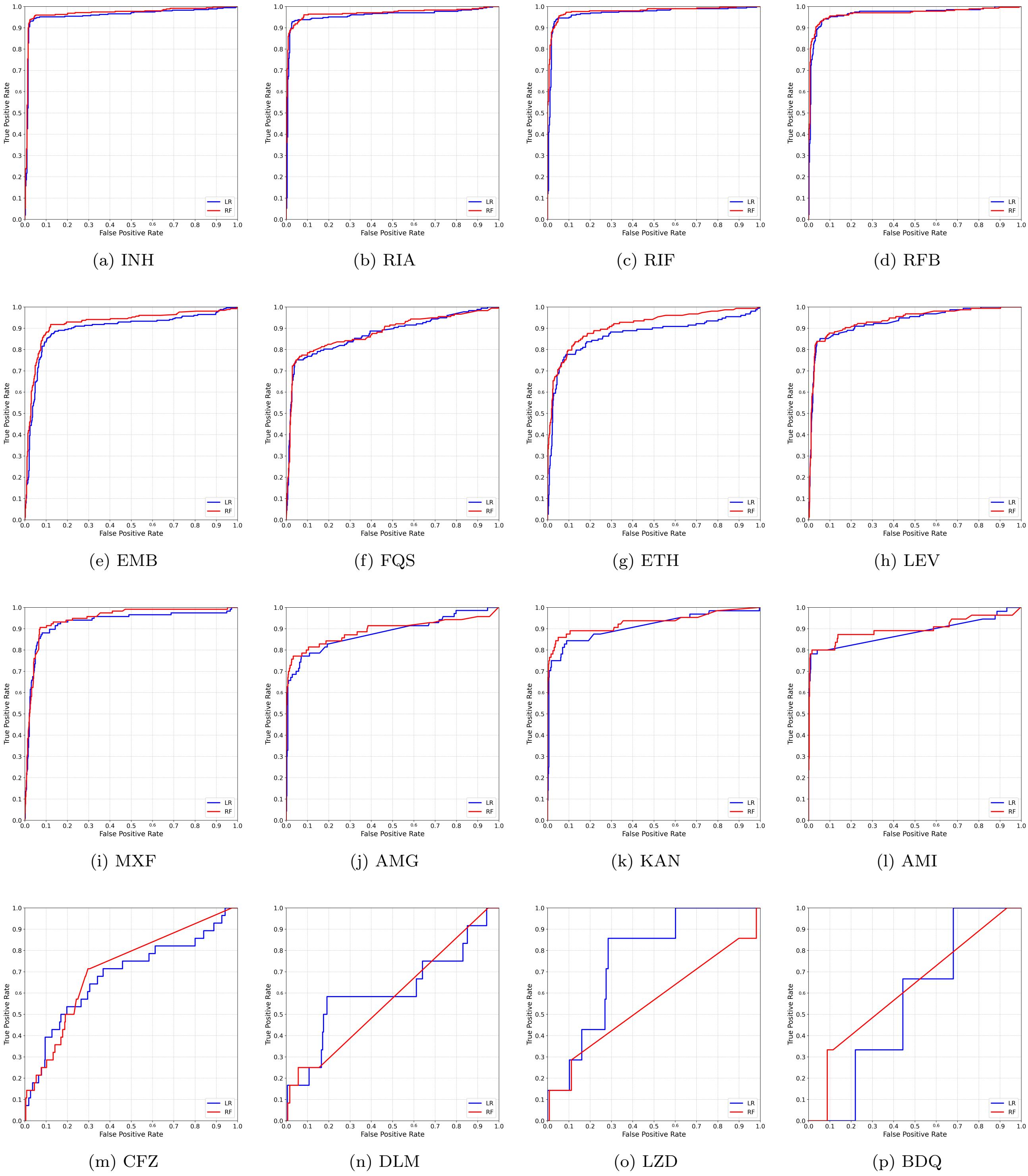

Fig. 4. ROC curve of the classification models for each resistance phenotype. The red color is related to RF and the green color is related to LR. The best LR and RF for each drug are highlighted in dark red and green.

Antibiotic drug	MEGARes Resistance Class	MEGARes Accession
AMG	Aminoglycoside	MEG_3, MEG_5, MEG_9, MEG_11, MEG_12, MEG_8078
	Rifampin	MEG_6090 , MEG_6134
	MTB-specific	MEG_3446
Kanamycin	Aminoglycoside	MEG_3, MEG_5, MEG_9, MEG_11, MEG_8078
	Rifampin	MEG_6090 , MEG_6134
	MTB-specific	MEG_3446
	FQS	MEG_3237
Amikacin	Aminoglycoside	MEG_5, MEG_9, MEG_11
Delamanid	Drug and Biocide Resistance	**MEG_3759**
	Multi-drug Resistance	**MEG_4078**
Linezolid	Oxazolidinone	**MEG_8670**
	Cationic AMR Peptides	MEG_1490

Table 6. Mechanisms of resistance found in the MEGARes database using BWA for all antibiotic drugs where the balance of the data is at most 11%. The bold genes in the table are uniquely found for that specific class of resistance.

Antibiotic Drug	MTB Gene
Isoniazid	**gyrA**, **rpoB**, **rpsL**, **fabG1**, **katG**, **embB**, rrs
Rifampicin	**rpoB**, **rpoC**, **rpsL**, **fabG1**, **katG**, **relA**, **Rv3327**, **dxs2**, **guaA**, **embB**, gyrA, rrs, mycP5, Rv2492, Rv2522c, fas, Rv3401, PE_PGRS57
Rifabutin	**rpoB**, **rpoC**, **mprA**, **katG**, **Rv2650c**, **cysA3**, **embB**, gyrA, Rv0376c, fadD30, PE_PGRS6, Rv0538, recC, rpsL, PE_PGRS13, kdpD, Rv1313c, rrs, PE_PGRS28, fabG1, wag22, PE_PGRS57, Rv3798
Ethambutol	**gyrA**, **rpoB**, **rpsL**, **fabG1**, **katG**, **dxs2**, **embB**, pknB, ctpA, Rv0095c, eccD3, dnaK, mmpL1, fadD30, rpoC, glpX, rrs, Rv1896c, Rv1897c, Rv2433c, obg, Rv2472, Rv3272, pcd, nth, embC, gid
Ethnomadie	**gyrA**, **rpoB**, **rrs**, **fabG1**, **pncA**, **embB**, mtc28, fadD30, rpoC, rpsL, Rv0760c, Rv0845, metS, kdpD, moeY, Rv1772, katG, Rv1995, Rv2219A, eis, Rv2522c, PE_PGRS44, deoA, alr, Rv3430c, Rv3603c, Rv3770c, Rv3910
Levofloxacin	**gyrA**
Moxifloxacin	**gyrA**
Kanamycin	**gyrA**, **narU**, **mmaA4**, **rpoB**, **pgi**, **murA**, **rrs**, **pncA**, **glnE**, **eis**, **PPE42**, **ethA**, Rv0516c, Rv1129c, Rv1145, mbtN, lipO, ctpD, katG, embB
Amikacin	**rrs**
Delamanid	**gadB**, PE_PGRS2, accD2, PE_PGRS22, phoH2, PE_PGRS29, Rv1508c, mmpL12, dnaE1, bioA, PE_PGRS30, pyrG, fadD1, fas, Rv2908c, mtrA
Linezolid	**rplC**, PE_PGRS16, cbs, fas, efp
Clofazimine	Rv0443
Bedaquiline	PE_PGRS48, PE_PGRS57

Table 7. Genes identified by aligning the top 31-mers associated with each antibiotic drug against MTB reference genome (H37Rv). Genes corroborating with findings from The CRyPTIC Consortium (2022) study are highlighted in bold to signify concordance. The remaining genes listed for each antibiotic drug were identified by alignment with at least ten distinct 31-mers.

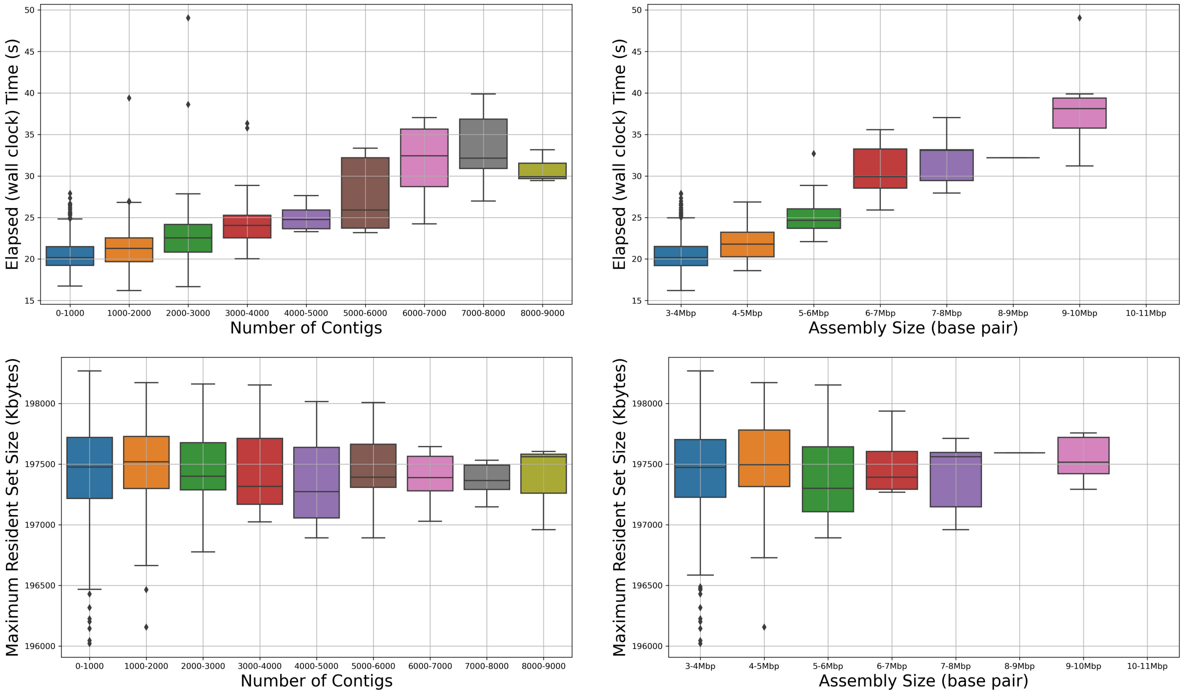

Fig. 5. Comparative Analysis of Genome Assembly Metrics and Computational Resources. Box plots illustrate the distribution of the number of contigs and the total assembly size across different isolates. Each metric is further analyzed in relation to peak memory usage and wall-clock time required for antibiotic drug resistance prediction using MTB++.
